# Lung cancer multi-omics digital human avatars for integrating precision medicine into clinical practice: the LANTERN study

**DOI:** 10.1186/s12885-023-10997-x

**Published:** 2023-06-13

**Authors:** Filippo Lococo, Luca Boldrini, Charles-Davies Diepriye, Jessica Evangelista, Camilla Nero, Sara Flamini, Angelo Minucci, Elisa De Paolis, Emanuele Vita, Alfredo Cesario, Salvatore Annunziata, Maria Lucia Calcagni, Marco Chiappetta, Alessandra Cancellieri, Anna Rita Larici, Giuseppe Cicchetti, Esther G.C. Troost, Róza Ádány, Núria Farré, Ece Öztürk, Dominique Van Doorne, Fausto Leoncini, Andrea Urbani, Rocco Trisolini, Emilio Bria, Alessandro Giordano, Guido Rindi, Evis Sala, Giampaolo Tortora, Vincenzo Valentini, Stefania Boccia, Stefano Margaritora, Giovanni Scambia

**Affiliations:** 1 Catholic University of the Sacred Heart, Rome, Italy; 2 Thoracic Surgery Unit, A. Gemelli University Hospital Foundation IRCCS, Rome, Italy; 3 Radiotherapy Unit, A. Gemelli University Hospital Foundation IRCCS, Rome, Italy; 4 Division of Oncological Gynecology, Department of Woman and Child Health and Public Health, A. Gemelli University Hospital Foundation IRCCS, Rome, Italy; 5 Departmental Unit of Molecular and Genomic Diagnostics, Genomics Core Facility, Gemelli Science and Technology Park (G-STeP), A. Gemelli University Hospital Foundation IRCCS, Rome, Italy; 6 Clinical Chemistry, Biochemistry and Molecular Biology Operations (UOC), A. Gemelli University Hospital Foundation IRCCS, Rome, Italy; 7 Medical Oncology, A. Gemelli University Hospital Foundation IRCCS, Largo A. Gemelli 8, Rome, Italy; 8 Open Innovation, Scientific Directorate, A. Gemelli University Hospital Foundation IRCCS, Rome, Italy; 9 CEO, Gemelli Digital Medicine & Health Srl, Rome, Italy; 10 Nuclear Medicine Unit, GsteP Radiopharmacy TracerGLab, A. Gemelli University Hospital Foundation IRCCS, Rome, Italy; 11 Institute of Pathology, A. Gemelli University Hospital Foundation IRCCS, Roma, Italy; 12 Advanced Radiodiagnostic Center, Department of Diagnostic Imaging, Oncological Radiotherapy and Hematology, A. Gemelli University Hospital Foundation IRCCS, Rome, Italy; 13 Department of Radiotherapy and Radiation Oncology, Faculty of Medicine and University Hospital Carl Gustav Carus, Technische Universität Dresden, Dresden, Germany; 14 Institute of Radiooncology – OncoRay, Helmholtz-Zentrum Dresden-Rossendorf, Rossendorf, Germany; 15 OncoRay – National Center for Radiation Research in Oncology, Faculty of Medicine and University Hospital Carl Gustav Carus, OncoRay - National Center for Radiation Research in Oncology, Technische Universität Dresden, Helmholtz-Zentrum Dresden-Rossendorf, Dresden, Germany; 16 German Cancer Consortium (DKTK), Partner Site Dresden, and German Cancer Research Center (DKFZ), Heidelberg, Germany; 17 National Center for Tumor Diseases (NCT), Partner Site Dresden, Germany: German Cancer Research Center (DKFZ), Heidelberg, Germany; Faculty of Medicine and University Hospital Carl Gustav Carus, Technische Universität Dresden, Dresden, Germany; Helmholtz Association / Helmholtz-Zentrum Dresden-Rossendorf (HZDR), Dresden, Germany; 18 ELKH-DE Public Health Research Group, Department of Public Health and Epidemiology, Faculty of Medicine, University of Debrecen, Debrecen, Hungary; 19 Institut de Recerca de l’Hospital de la Santa Creu i Sant Pau (IR-HSCSP), Barcelona, Spain; 20 School of Medicine, Turkey and Koç University Research Center for Translational Medicine (KUTTAM), Sariyer, Koç University, Istanbul, Turkey; 21 Department of Philosophy and Educational Sciences, University of Turin - Academy of the Expert Patient ADPEE - EUPATI, Turin, Italy; 22 Interventional Pulmonology Unit, A. Gemelli University Hospital Foundation IRCCS, Rome, Italy; 23 Department of Basic Biotechnological Sciences, Intensivological and Perioperative Clinics, Catholic University of Sacred Heart, Rome, Italy; 24 Department of Life Sciences and Public Health, Catholic University of Sacred Heart, Rome, Italy

**Keywords:** Lung cancer, Artificial intelligence (AI), Digital human avatars (DHA), Personalize medicine, Machine learning, System medicine, Precision medicine, Genomics, Radiomics, Big data

## Abstract

**Background:**

The current management of lung cancer patients has reached a high level of complexity. Indeed, besides the traditional clinical variables (e.g., age, sex, TNM stage), new omics data have recently been introduced in clinical practice, thereby making more complex the decision-making process. With the advent of Artificial intelligence (AI) techniques, various omics datasets may be used to create more accurate predictive models paving the way for a better care in lung cancer patients.

**Methods:**

The LANTERN study is a multi-center observational clinical trial involving a multidisciplinary consortium of five institutions from different European countries. The aim of this trial is to develop accurate several predictive models for lung cancer patients, through the creation of Digital Human Avatars (DHA), defined as digital representations of patients using various omics-based variables and integrating well-established clinical factors with genomic data, quantitative imaging data etc. A total of 600 lung cancer patients will be prospectively enrolled by the recruiting centers and multi-omics data will be collected. Data will then be modelled and parameterized in an experimental context of cutting-edge big data analysis. All data variables will be recorded according to a shared common ontology based on variable-specific domains in order to enhance their direct actionability. An exploratory analysis will then initiate the biomarker identification process. The second phase of the project will focus on creating multiple multivariate models trained though advanced machine learning (ML) and AI techniques for the specific areas of interest. Finally, the developed models will be validated in order to test their robustness, transferability and generalizability, leading to the development of the DHA. All the potential clinical and scientific stakeholders will be involved in the DHA development process. The main goals aim of LANTERN project are: i) To develop predictive models for lung cancer diagnosis and histological characterization; (ii) to set up personalized predictive models for individual-specific treatments; iii) to enable feedback data loops for preventive healthcare strategies and quality of life management.

**Discussion:**

The LANTERN project will develop a predictive platform based on integration of multi-omics data. This will enhance the generation of important and valuable information assets, in order to identify new biomarkers that can be used for early detection, improved tumor diagnosis and personalization of treatment protocols.

**Ethics Committee approval number:**

5420 − 0002485/23 from Fondazione Policlinico Universitario Agostino Gemelli IRCCS – Università Cattolica del Sacro Cuore Ethics Committee.

**Trial registration:**

clinicaltrial.gov - NCT05802771.

## Background

The current care strategy for lung cancer patients still relies on decisional mechanisms based on traditional clinical variables (e.g., age, sex, TNM stage) that are insufficient to deal with such a complex disease, especially considering the exponential growth of new actionable biological variables that characterized the last decade [1]. In recent times, the advent of genomics and precision medicine has indeed transformed the horizon, for scientists, physicians and patients. This ongoing revolution of personalize medicine has accelerated the combined efforts of many researchers to define molecular subgroups of lung cancer, characterize the genomic landscape of lung cancer subtypes, identify novel therapeutic targets and understand the mechanisms of sensitivity and resistance to targeted therapies [2]. The clinical implementation of these results can now be observed in lung cancer, patients receiving molecular tests to determine whether their tumours contain actionable mutations, improved targeted therapies able to overcome resistance to first-generation drugs and drugs targeting the immune system being currently tested in clinical trials. In addition, the characterization of individuals’ phenotypes and genotypes (e.g., molecular profiling, medical imaging and lifestyle data) are also aiding prevention strategies as well as tailoring the right therapeutic path to take at the right time [3]. The information obtained from the recalled - omics technologies provide physicians with an enormous amount of data (“big data”). Apart from analysing and interpreting these data, the task of integrating them into health systems still seems too complex for standard decision-making mechanisms. Conversely, with the application of Artificial Intelligence (AI) techniques, researchers now have the potential to deal with such problems. Artificial intelligence models aid the recognition of complex patterns in medical data, composed by heterogeneous data sets and diverse omics profiles, and provide robust predictions for several clinical conditions and outcomes [4, 5] as reported below in detail. Therefore, the main hypothesis of the “LANTERN” project is to implement of precision medicine in lung cancer, through the creation of DHA which will serve as accurate predictive models for lung cancer patients, using prospective multi-omics data to support clinicians in improving decision-making processes. This manuscript describes the design, methodology and the expected results of this study.

## Methods/design

The LANTERN project aims to deliver a novel approach for comprehensive lung cancer decision making solutions, based on predictive digital platforms powered by the integration multi-omics data. The use of such platform will allow the development of innovative technological prototypes, improving the accuracy of diagnosis and offering patients a complete personalization of their oncological treatment.

LANTERN is a multi-centric observational clinical trial involving a multinational and interdisciplinary collaboration among five oncological research institutions and a private patient organization.

**Fig. 1:**
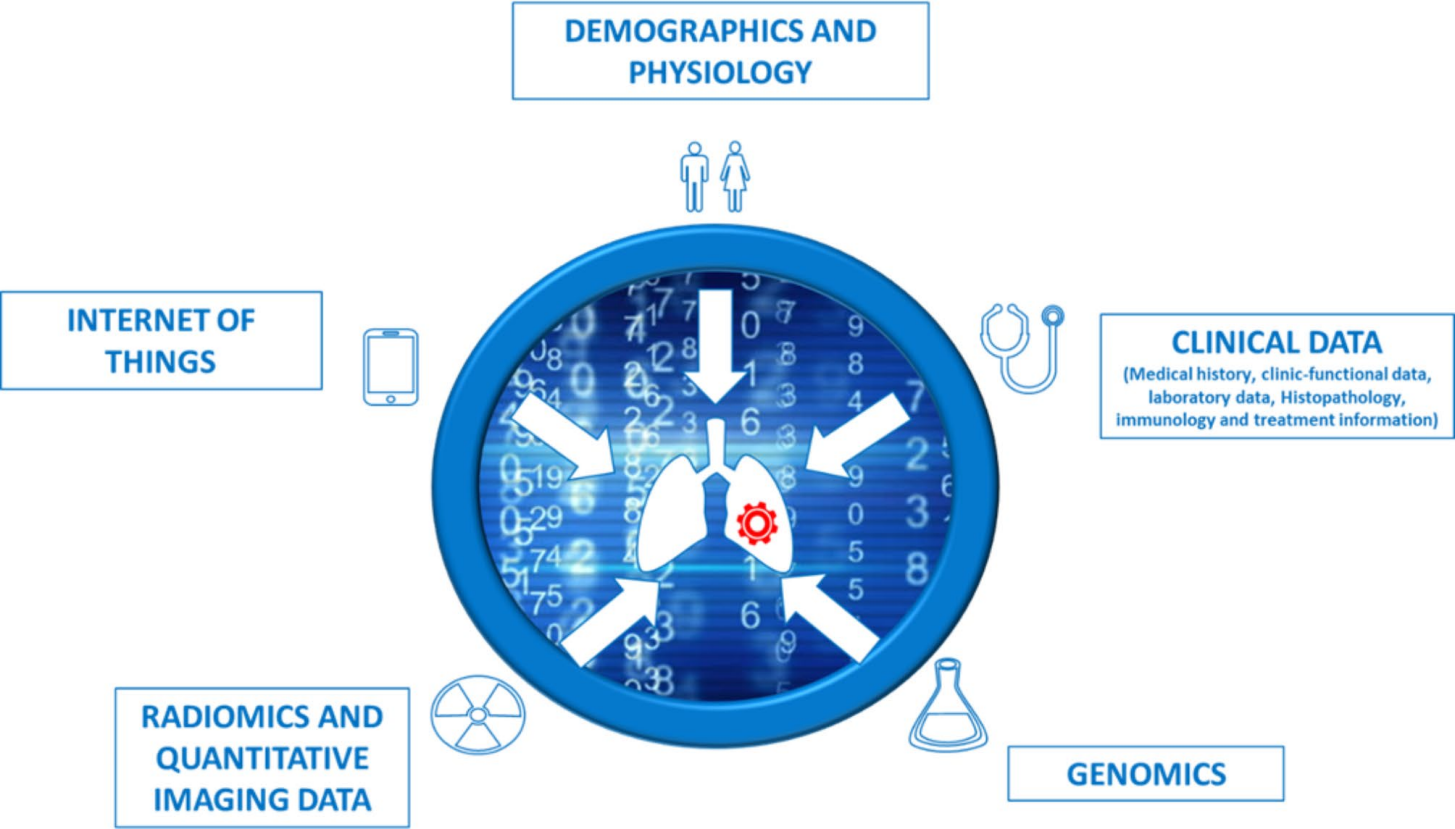
This figure illustrates the five omics-based variables which encompass all data to be collected for the purpose of this study

The consortium partners include: the coordinating site, Fondazione Policlinico Universitario “A. Gemelli” IRCCS, Italy (FPG); Technische Universität Dresden, Germany (TUD); University of Debrecen, Hungary (DEB); Hospital de la Santa Creu i Sant Pau, Catalona Spain (HSCSP); Koç University School of Medicine, Turkey (KU) and EUPATI Expert Patient Academy (AdPEE) (patient organization). Five omics-based domains will be considered in this study, reflecting all the omics-domains involved in the lung cancer decision making process and encompassing all data to be collected from the enrolled patients as summarized herein and in Fig. 1:

A. Demographic and physiological data.
B. Clinical data.
C. Genomics and Proteomics.
D. Radiomics and quantitative imaging data.
E. Internet of Things derived data.

### Methodology

The LANTERN project has been funded by the ERA PerMed JTC2022 (Proposal ERAPERMED2022-116) and will be completed within 36 months.

**Fig. 2:**
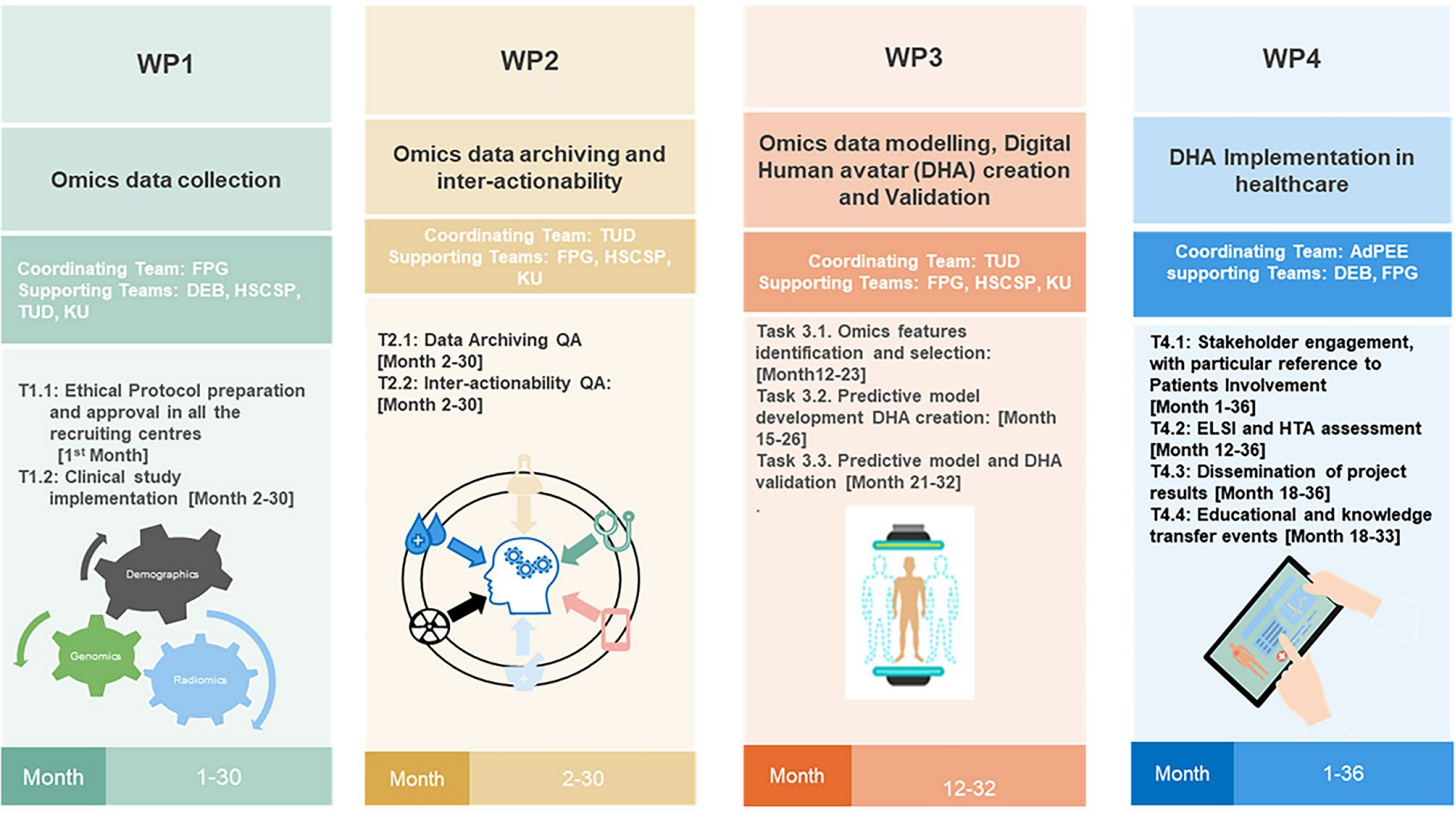
This table shows the distribution of the work packages among the consortium partners and their respective timelines

The project has been divided into four work packages (WP) with specific timelines and tasks which will be distributed among the consortium partners (Fig. 2):

WP1: Patient enrollment and omics data collection [Months 1–30].

WP2: Omics data archiving and inter-actionability [Months 2–30].

WP3: Omics data modelling, Digital avatar (DHA) creation and validation [Months 12–32].

WP4: DHA implementation in healthcare [Months 1–36].

### WP1: patient enrolment and omics data collection

The objective of WP1 is to gather information from all the clinical and omics-based data sources currently considered as clinically significant for decision support in lung cancer comprehensive diagnosis and therapy workflow. A structured terminology data collection system will be developed for prospective data collection through specific Case Report Forms (CRFs). Patients will be enrolled by the clinical research enrolment centres and data obtained from the five omics-based variables (Fig. 1) will be collected and stored in a secure database. This WP represents the initiation process of the study which also involves the Ethical Protocol preparation and approval in all the recruiting centres which will be carried out within the first month of the activities. Following the protocol approval in all centres, patients that are considered eligible will start to be enrolled.

### WP2: omics data archiving and inter-actionability

The main aim of this WP is to allow complete data integration into both existing and new archiving systems and to ensure an easy and effective use and sharing of collected omics data. All collected data representing the different considered omics-domains will be recorded according to a shared common ontology. All the variables for nomenclature and units of measurements will be standardized to achieve uniformity among the datasets. The shared general ontology will represent a structured terminological system for data archiving and analysis where all the different omics domains will be recorded in a specific eCRF, ensuring coherence for all the collected data variables. Possible discrepancies originating from the use of different technical systems for data capture will be solved through a unanimous decision made by the research teams. In addition, all the laboratory data indicated in WP1 will undergo cross quality assurance (QA) procedures prior to data upload in the shared platform.

### WP3: omics data modelling, digital human avatar (DHA) creation and validation

This WP is focused on developing accurate predictive models and their validation. The purpose of this WP is to identify predictive biomarkers, harmonize them through compact statistical models and subsequently create patient specific DHAs. We plan to integrate all the aforementioned omics data into predictive models that will represent the basis for a fully personalized and innovative lung cancer integrated decision support system.

This WP is divided into three phases:

Phase 1: Omics feature identification and selection.

Phase 2: Predictive model development and DHA creation.

Phase 3: Predictive model and DHA validation.

#### Omics features identification and selection

In the first step, an exploratory analysis across all collected datasets from an estimate of ≈ 240 NSCLC patients will enable the start of the identification process of a more selected pool of stable and promising potential biomarkers.

We will apply robust data analysis techniques in order to identify relevant variables in a univariate setting, taking individual statistical distributions, feature-relevant correlations and general descriptive statistics into account.

#### Predictive model development and DHA creation

The objective of the second phase is to create multiple modular multivariate models which will be trained using ML algorithms, and the subsequent creation of the DHA. Different supervised models will be developed including logistic regression, decision tree, support vector machine, random forest, XGBoost classifier, and artificial neural networks as already reported in the literature [6–12]. The k-fold cross-validation will be used for hyperparameters tuning and statistical significance comparison of the performance of the ML models will be then evaluated.

The DHA creation will involve the integration of specific algorithms into the data extraction pipeline to clean and restructure the flow of data. The results of this pre-processing will then be recoded through a specifically assigned ontology to reveal duplicates. This will lead to the creation of Data Marts which will be updated continuously and automatically with new data. Based on the available already processed data, the developed algorithm and its underlying infrastructure will be used to classify newly updated patient data inputs by the clinicians using the interface. The resulting data presented through the dynamic interface allows the thorough exploration of previously added patient data already present in the database, to infer the best course of action based on historical data and the experience of the clinician.

Both user friendliness and model explainability will serve as the primary standard of the model development strategies. Easily interpretable values such as SHAP (SHapley Additive exPlanations) values will be attached to each model in order to avoid any black-box approaches that might render model outputs difficult to explain to the patients during their interactions with the clinicians.

#### Predictive model and DHA validation

Both the developed model and the comprehensive DHA will be validated in order to test their robustness, transferability and generalizability. Two consecutive validation steps will be employed respectively: the internal and external validation. We estimate a total number of approximately 420 NSCLC cases to start the validation process. The internal validation step will be focused mainly on techniques such as K-fold cross validation. This will allow to achieve a Transparent Reporting of a multivariable prediction model for Individual Prognosis or Diagnosis (TRIPOD) level 2b [13]. At this TRIPOD level, data are non-randomly split (e.g. by location or time) into two groups: one for the training process and the remaining dataset to test its predictive performance. In the case of suboptimal performances, all the failing models will be reverted back to the previous stage and the models will be retrained with new specifications in order to increase their robustness. At this point, validation can be done using external datasets, reaching TRIPOD 3 and 4 levels (external validation). Finally, when a TRIPOD 4 level is reached, data transferability and full actionability will be granted, allowing the successful and safe application of the model in the clinical routine.

### WP4: DHA implementation in healthcare

The objective of this WP is to ensure the sustainable and organized implementation of the validated DHA in clinical practice by engaging all the involved stakeholders (i.e., patients, clinicians, clinical and computer engineers, statisticians, health economists, bioethicists, decision makers, researchers etc.). The data provided from the project will be used within the EUnetHTA core model [EUnetHTA Joint Action 2, WP 8. HTA Core Model v3.0; 2016] to take into consideration the essential aspects for the proper implementation of the DHA in healthcare, such as the clinical, epidemiological, technical, economic, organizational, ethical, social and legal implications. Organization of tailored communication activities for decision makers, national and international health authorities and publicity strategies (press releases, webinars) will be initiated with special focus in patient engagement through the help of the patient organisation (ADPEE). Finally, the interim and final results of the projects will be published in peer-reviewed indexed scientific international journals, scientific information platforms, patient’s advocacy and association websites.

### Data management plan

A well-structured Data Management Plan (DMP) for LANTERN project will be developed in the third month after the start of the project. The DMP will describe the data management life cycle for the data to be collected, processed, generated, preserved, and re-used both during and after the end of the LANTERN project. During the project, the focus of data management will be on complying with the General Data Protection Regulation (GDPR (European Union, 2016)) for privacy-abiding treatment of health data. Towards the end of the project, priority will be given to making the data as openly accessible as possible, but at the same time, strictly monitored. This project is committed to allow the reuse of research data according to the principles of findability, accessibility, interoperability and reusability (FAIR) to make the data findable, accessible, interoperable, and reusable. In addition to the usual clinical and biological data, a customized application (Healthentia) synchronized with smartphones or wearables (e.g., fitness bracelets), will be used to collect real world data (RWD), such as physical activity, sleep and vital signs (i.e., heart beats). Such data will be collected from the prospective study that will start in the second month of the project. All patients will be provided with detailed information on the study and will be requested to sign an informative consent form. Data collection will be realized using REDCap, a web application fully compliant with the 21 CFR Part 11 and GDPR. CRFs will be designed according to GCP and GLP requirements, the development will include annotated CRF design according to protocol, database setup and its validation.

A remote monitoring system will be put in place during data collection to manage discrepancies and inconsistencies and generate queries. All data should be ALCOA + criteria compliant. Furthermore, the project LANTERN will strictly follow privacy- and security-by-design principles and use highly innovative secure multi-party computation techniques to satisfy all security and privacy-related requirements imposed by the legal framework and ethical considerations of its users. In particular, the system will be accessed from multiple sites by dedicated researchers through a two-step verification login process and an auto-logout, to ensure data protection. An audit will be set up to automatically monitor all user activities. The database will be GDPR compliant by design and the data will be pseudonymized, a code will be assigned to each patient to allow data linkage. The corresponding patient codes, representing their identity will be stored by the principal investigator of the project. The prospective dataset provided by the clinical partners (FPG, TUD, KU, HSCSP) will only be accessible to the partners as defined by the data processing agreement. Finally, project results (mainly research publications, project deliverables, research datasets and predictive models), LANTERN will adopt Open Science Practices, fostering their accessibility.

### Eligibility criteria

Patients older than 18 years of age with pathologically proven diagnosis of non-small cell lung cancer (NSCLC) will be considered eligible for this study. No gender or disease stage filters will be applied at selection, to ensure an adequate representation of NSCLC patient population and to avoid any form of bias. Sample size was calculated on the basis of G-power R program for “a priori” analysis, considering α = 5% and power of 90% and an estimate of 600 patients has been deemed sufficient to power the analysis.

### Statistical analysis

For all the considered omics domains, the Wilcoxon Mann-Whitney test will be used to investigate the ability of identified candidate biomarkers (“features”) to predict clinical outcomes on univariate analysis. The feature showing the lowest p-value will be considered as the most predictive parameter and selected for multiple variable tests. Receiver Operating Feature curve analysis (ROC) will also be performed on the most significant feature obtained at WMW analysis, calculating the area under the curve (AUC), with the corresponding 95% confidence interval (CI) according to the Clopper-Pearson method. Model processing will be performed through advanced ML and AI techniques, firstly on a dedicated training dataset to fit the model parameters. The so obtained hyper-parameters will be tuned on a dedicated validation dataset and finally an independent dataset with the same probability distribution will be employed as validation set, stressing the replicability of the set model.

### Ethics aspects of the project

The main ethical issues related to this project include: *(i) the respect for persons and for human dignity; (ii) fair distribution of benefits and burden; (iii) the rights and interests of the participants; (iv) the need to ensure participants’ informed consent; (v) processing of personal data; (vi) concerns involving the development, deployment and/or use of artificial intelligence (AI)-based systems or techniques.*

Therefore, the LANTERN partners will identify and implement a comprehensive set of ethical and legal requirements in accordance with Declaration of Helsinki and GDPR principles to ensure compliance for all activities of the project. Appropriate ethical monitoring systems will also be set up during all the phases of the project.

## Discussion

The current approach to lung cancer is driven by an acute and episodic model of care (so-called “reactive medicine”), which is rapidly reaching its limit and becoming economically unsustainable, especially in the context of over-stressed national health systems [14]. This project will offer an integrated multi-omics platform for the management of lung cancer patients that will optimize the financial and human resources of the healthcare system. Indeed, the development of DHA will provide a more efficient and targeted system of treatment and prevention in these patients [15, 16]. A significant reduction in healthcare costs, access to care and social inequalities are therefore expected in terms of reduction of patient visit times and costs of overtreatment; reduction of costs for toxicity treatment; faster recovery times and reduction of overall hospitalization times; access to rapid and adequate healthcare services by all patients irrespective of age, sex and social status. The development of DHA and the consequent application of predictive models in clinical practice will also improve the accuracy of diagnosis and will facilitate a complete personalization of the treatment [17, 18]. In summary, patients facing better, more effective and personalized treatment pathways will be able to see the duration of therapies reduced with positive impacts both in terms of reduction of direct costs (in terms of number of sessions, of trips in the case of services performed off-site, ancillary examinations, etc.) and indirect (i.e., a better state of health). In addition, the smart data linkage (i.e., linking behavioural, lifestyle, environmental and omics data) will also be a catalyst to change the relationship between individuals and their health thus resulting in a significant cultural and societal impact. Indeed, the collection and availability of personal wellbeing information (provided by health portable devices – “Internet of Things”) will not only enable the development of more efficient interventions in lung cancer, but also empowers patients to improve their own life experience (e.g., improved diet, exercise, or other lifestyle choices). Furthermore, the prospective technology solutions enabled by our project will also benefit the MedTech, Data Analytics and Pharmaceutical industries in creating new opportunities for existing businesses and boosting new entrants to provide value-based solutions.

## Data Availability

In terms of data managed during project implementation, LANTERN will collect and store data from 600 NSCLC patients from four EUROPEAN oncological institutions (FPG, TUD, KU, HSCSP). Data collection will be performed using electronic CRFs exclusively. CRFs will be designed according to GCP requirements, the development will include annotated CRF design according to protocol, database setup and validation, edit checks programming. Data collection will be realized using the REDCap platform, a web application fully compliant with the 21 CFR Part 11 and GDPR. Results obtained from the LANTERN project will be open access.

## Abbreviations

NSCLC: Non-small cell lung cancer
AI: Artificial intelligence
DHA: Digital Human Avatars
ML: Machine learning
WP: Work packages
TNM: Tumour, Node, Metastases
CRF: Case Report Form
QA: Quality assurance
SHAP: Shapley Additive explanations
TRIPOD: Transparent reporting of a multivariable prediction model for individual prognosis or diagnosis
GDPR: General Data Protection Regulation
GCP: Good Clinical Practice
GLP: Good Laboratory Practice
ROC: Receiver operating characteristic curve
AUC: Area under curve
CI: Confidence interval
