# Correction: Lung cancer multi-omics digital human avatars for integrating precision medicine into clinical practice: the LANTERN study

**DOI:** 10.1186/s12885-023-11606-7

**Published:** 2023-11-09

**Authors:** Filippo Lococo, Luca Boldrini, Charles-Davies Diepriye, Jessica Evangelista, Camilla Nero, Sara Flamini, Angelo Minucci, Elisa De Paolis, Emanuele Vita, Alfredo Cesario, Salvatore Annunziata, Maria Lucia Calcagni, Marco Chiappetta, Alessandra Cancellieri, Anna Rita Larici, Giuseppe Cicchetti, Esther G. C. Troost, Róza Ádány, Núria Farré, Ece Öztürk, Dominique Van Doorne, Fausto Leoncini, Andrea Urbani, Rocco Trisolini, Emilio Bria, Alessandro Giordano, Guido Rindi, Evis Sala, Giampaolo Tortora, Vincenzo Valentini, Stefania Boccia, Stefano Margaritora, Giovanni Scambia

**Affiliations:** 1 Catholic University of the Sacred Heart, Rome, Italy; 2 Thoracic Surgery Unit, A. Gemelli University Hospital Foundation IRCCS, Rome, Italy; 3 Radiotherapy Unit, A. Gemelli University Hospital Foundation IRCCS, Rome, Italy; 4 Division of Oncological Gynecology, Department of Woman and Child Health and Public Health, A. Gemelli University Hospital Foundation IRCCS, Rome, Italy; 5 Departmental Unit of Molecular and Genomic Diagnostics, Genomics Core Facility, Gemelli Science and Technology Park (G-STeP), A. Gemelli University Hospital Foundation IRCCS, Rome, Italy; 6 Clinical Chemistry, Biochemistry and Molecular Biology Operations (UOC), A. Gemelli University Hospital Foundation IRCCS, Rome, Italy; 7 Medical Oncology, A. Gemelli University Hospital Foundation IRCCS, Largo A. Gemelli 8, Rome, Italy; 8 Open Innovation, Scientific Directorate, A. Gemelli University Hospital Foundation IRCCS, Rome, Italy; 9 CEO, Gemelli Digital Medicine & Health Srl, Rome, Italy; 10 Nuclear Medicine Unit, GsteP Radiopharmacy TracerGLab, A. Gemelli University Hospital Foundation IRCCS, Rome, Italy; 11 Institute of Pathology, A. Gemelli University Hospital Foundation IRCCS, Rome, Italy; 12 Advanced Radiodiagnostic Center, Department of Diagnostic Imaging, Oncological Radiotherapy and Hematology, A. Gemelli University Hospital Foundation IRCCS, Rome, Italy; 13 Department of Radiotherapy and Radiation Oncology, Faculty of Medicine and University Hospital Carl Gustav Carus, Technische Universität Dresden, Dresden, Germany; 14 Institute of Radiooncology – OncoRay, Helmholtz-Zentrum DresdenRossendorf, Rossendorf, Germany; 15 OncoRay – National Center for Radiation Research in Oncology, Faculty of Medicine and University Hospital Carl Gustav Carus, OncoRay - National Center for Radiation Research in Oncology, Technische Universität Dresden, Helmholtz-Zentrum Dresden-Rossendorf, Dresden, Germany; 16 German Cancer Consortium (DKTK), Partner Site Dresden, and German Cancer Research Center (DKFZ),, Heidelberg, Germany; 17 National Center for Tumor Diseases (NCT), Partner Site Dresden, Germany: German Cancer Research Center (DKFZ), Heidelberg, Germany; Faculty of Medicine and University Hospital Carl Gustav Carus, Technische Universität Dresden, Dresden, Germany; Helmholtz Association / Helmholtz-Zentrum Dresden-Rossendorf (HZDR), Dresden, Germany; 18 ELKH-DE Public Health Research Group, Department of Public Health and Epidemiology, Faculty of Medicine, University of Debrecen, Debrecen, Hungary; 19 Institut de Recerca de L’Hospital de La Santa Creu I Sant Pau (IR-HSCSP), Barcelona, Spain; 20 School of Medicine, Turkey and Koç University Research Center for Translational Medicine (KUTTAM), Sariyer, Koç University, Istanbul, Turkey; 21 Department of Philosophy and Educational Sciences, University of Turin - Academy of the Expert Patient ADPEE - EUPATI, Turin, Italy; 22 Interventional Pulmonology Unit, A. Gemelli University Hospital Foundation IRCCS, Rome, Italy; 23 Department of Basic Biotechnological Sciences, Intensivological and Perioperative Clinics, Catholic University of Sacred Heart, Rome, Italy; 24 Department of Life Sciences and Public Health, Catholic University of Sacred Heart, Rome, Italy

**Correction: BMC Cancer 23, 540 (2023)**

**https://doi.org/10.1186/s12885-023-10997-x**

Following publication of the original article [1], the authors identified an error in the author name of Róza Ádány. The given name and family name were erroneously transposed.

The incorrect author name is: Ádány Róza

The correct author name is: Róza Ádány

The author group has been updated above and the original article [1] has been corrected.

## References

[1] Lococo F, Boldrini L, Diepriye CD (2023). Lung cancer multi-omics digital human avatars for integrating precision medicine into clinical practice: the LANTERN study. BMC Cancer.

